# Supplementary material for: Clinical profile, prognosis and post COVID-19 syndrome among UNRWA staff in Jordan: A clinical case-series study
Source: PLoS One. 2023 Nov 9;18(11):e0293023. doi: 10.1371/journal.pone.0293023 (PMC10635548; doi:10.1371/journal.pone.0293023)
Supplement: Attachment — Submitted filename: Response to Reviewers.docx [file pone.0293023.s002.docx]

**Re: Clinical Profile, Prognosis and Post COVID-19 Syndrome among UNRWA Staff in Jordan: A Clinical Case-Series Study**

Dear Reviewers,

I hope this letter finds you well. First and foremost, I would like to express my gratitude for taking the time to review my publication. Your constructive feedback and comments have been invaluable in improving the quality and clarity of my work.

I would like to address the concerns and comments raised in your review. I have carefully considered each one of them and have made the necessary changes to the manuscript accordingly.

So please find the following comments and responses.

**Line 58 - "was reported in Wuhan"**

Changed according to reviewer comment.

**Line 66 - "differs"**

Changed according to reviewer comment.

**Lines 68 - 71 - several studies have now explored Long COVID. Are there any from Jordan, or closer in the region, that would allow for more country- / region-specific comparison? You mention no such studies, but I had a quick look and saw this pre-print paper: https://www.emro.who.int/in-press/research/post-covid-syndrome-among-jordanian-health-care-workers.html, and this: xhttps://www.iproc.org/2022/1/e36563/:**

This was reviewed now and justified on the manuscript, in addition to this pre-print we found another study also.

**Line 77 - 78 - is this also a study of risk factors?**

No it is not, we made the correction on the manuscript.

**Line 93 - as Reviewer 1 has mentioned, this requires elaboration. The sample frame is clear - i.e. all known positive staff (presumably lab tested? - perhaps you can also elaborate how a positive diagnosis was determined). But the sampling strategy is not. How were these people selected?**

Sample strategy is now clarified in more details on the manuscript.

**Line 93 - 94 - might be worth explaining why those with a recent diagnosis were excluded - perhaps a definition of long COVID earlier in the manuscript.**

Exclusion criteria according to post COVID definition is now clarified on the manuscript.

**Line 107 - can you elaborate what the pilot study involved? How was validation achieved?**

We explained more about the pilot study now on the manuscript.

**Line 233 - 237 - some negative findings might be worth presenting. Did smoking status have no other statistically significant impact on other symptoms? Severity of illness, SOB, etc?**

Some related negative findings added.

**Line 265 - "older people over the age of 60"**

Changed according to reviewer comment.

**Line 271 - how were asymptomatic people identified? Did UNRWA have a testing system in place at that time?**

According to our country policy at that time, any person who had a contact with infected person should do the PCR test, so some employees contacted infected people while they are free of symptoms. Staff who were contacting their infected symptomatic relatives, were reporting this to their work in which they will be asked to conduct quarantine and testing accordingly. So they inform UNRWA and being part of its record of infected employees

**Line 285 - I don't think there's any strong basis with which to suggest symptom profiles differed by race and ethnicity**

This is removed from the Manuscript-Not Justified and we agree with the reviewer.

**Line 319 - 320 - here you mention a Jordan-based long COVID study, which runs counter to your earlier suggestion of no pre-existing country research**

We added the pre-print and the study that we found now earlier in the manuscript.

**Line 372 - people of working age (broad as that is 18-65ish) can have high rates of HTN, T2DM - how does this compare to the general Jordanian population?**

We compare this now with the general population figures.

**Line 388 - again, how this is a representative sample needs to be elaborated, as the sampling strategy is unclear**

We elaborate on sample selection in the methods section earlier now.

**Line 395 - a major limitation here is that this study didn't have a longitudinal dimension. So symptoms reported have been attributed to COVID, but we don't know if people had pre-existing fatigue, forgetfulness, depression, etc.**

According to the definition of post COVID19 syndrome WHO, Symptoms were considered positive only if persisted for 2 months or more after 12 weeks from date of infection and the symptom cannot be explained by an alternative diagnosis. we focused when we conducted the questionnaire that the symptom was newly developed and no other diagnosis explained it, otherwise it would be considered negative.

**Line 402 - Conclusion should not reiterate results, but take your Discussion and wrap-up with next steps, what this adds, why it makes any difference, who should take note, etc.**

The conclusion section is now modified according to your advice.

**Regarding the Journal Requirements:**

1. Please ensure that your manuscript meets PLOS ONE's style requirements, including those for file naming. The PLOS ONE style templates can be found at 
<https://journals.plos.org/plosone/s/file?id=wjVg/PLOSOne_formatting_sample_main_body.pdf> and 
<https://journals.plos.org/plosone/s/file?id=ba62/PLOSOne_formatting_sample_title_authors_affiliations.pdf>

**Manuscript submitted according to these requirements**

2. Thank you for stating the following financial disclosure: 
"No"

At this time, please address the following queries:

a) Please clarify the sources of funding (financial or material support) for your study. List the grants or organizations that supported your study, including funding received from your institution. :

**The researcher conducted the study during her duty working hours as part of the COVID-19 response team.**

b) State what role the funders took in the study. If the funders had no role in your study, please state: “The funders had no role in study design, data collection and analysis, decision to publish, or preparation of the manuscript.”

**The funders had no role in study design, data collection and analysis, decision to publish, or preparation of the manuscript**

c) If any authors received a salary from any of your funders, please state which authors and which funders.

**NA**

d) If you did not receive any funding for this study, please state:

“**The authors received no specific funding for this work.”**

Please include your amended statements within your cover letter; we will change the online submission form on your behalf.

3. Thank you for stating the following in your Competing Interests section:  
"No"

Please complete your Competing Interests on the online submission form to state any Competing Interests. If you have no competing interests, please state as detailed online in our guide for authors at <http://journals.plos.org/plosone/s/submit-now> . This information should be included in your cover letter; we will change the online submission form on your behalf.

**"The authors have declared that no competing interests exist.as none of the authors has financial or personal interests that may influence the research findings”**

4. In your Data Availability statement, you have not specified where the minimal data set underlying the results described in your manuscript can be found. PLOS defines a study's minimal data set as the underlying data used to reach the conclusions drawn in the manuscript and any additional data required to replicate the reported study findings in their entirety. All PLOS journals require that the minimal data set be made fully available. For more information about our data policy, please see <http://journals.plos.org/plosone/s/data-availability.-->

**Data Attached in an Excel File**

"Upon re-submitting your revised manuscript, please upload your study’s minimal underlying data set as either Supporting Information files or to a stable, public repository and include the relevant URLs, DOIs, or accession numbers within your revised cover letter. For a list of acceptable repositories, please see <http://journals.plos.org/plosone/s/data-availability#loc-recommended-repositories>. Any potentially identifying patient information must be fully anonymized.

Important: If there are ethical or legal restrictions to sharing your data publicly, please explain these restrictions in detail. Please see our guidelines for more information on what we consider unacceptable restrictions to publicly sharing data: <http://journals.plos.org/plosone/s/data-availability#loc-unacceptable-data-access-restrictions>. Note that it is not acceptable for the authors to be the sole named individuals responsible for ensuring data access.

We will update your Data Availability statement to reflect the information you provide in your cover letter.

5. PLOS requires an ORCID iD for the corresponding author in Editorial Manager on papers submitted after December 6th, 2016. Please ensure that you have an ORCID iD and that it is validated in Editorial Manager. To do this, go to ‘Update my Information’ (in the upper left-hand corner of the main menu), and click on the Fetch/Validate link next to the ORCID field. This will take you to the ORCID site and allow you to create a new iD or authenticate a pre-existing iD in Editorial Manager. Please see the following video for instructions on linking an ORCID iD to your Editorial Manager account: <https://www.youtube.com/watch?v=_xcclfuvtxQ>

**ORCID ID Provided**

6. Please include your full ethics statement in the ‘Methods’ section of your manuscript file. In your statement, please include the full name of the IRB or ethics committee who approved or waived your study, as well as whether or not you obtained informed written or verbal consent. If consent was waived for your study, please include this information in your statement as well.

**Ethical statement mentioned in Methodology section**

Once again, I would like to thank you for your time and effort in reviewing my publication. Your insights and suggestions have been invaluable, and I believe that they have greatly improved the quality of my work. If you have any further comments or suggestions, please do not hesitate to let me know.

Sincerely,

Haneen Aldahleh
