# Supplementary material for: Clinical profile, prognosis and post COVID-19 syndrome among UNRWA staff in Jordan: A clinical case-series study
Source: PLoS One. 2023 Nov 9;18(11):e0293023. doi: 10.1371/journal.pone.0293023 (PMC10635548; doi:10.1371/journal.pone.0293023)
Supplement: Attachment — Submitted filename: Response to Reviewers 2.docx [file pone.0293023.s003.docx]

1. **you mentioned that 1322 of the staff developed COVID-19 while your sample size is only 366, I believe these are your colleagues and easily reachable, therefore why did you limit the sample to quarter of the eligible participants.**

The decision to limit our sample size was based on both scientific considerations and practical logistics. it might seem straightforward to engage with colleagues for data, the constraints of resources, combined with the detailed nature of data collection – which included not just the time of the researcher but also the expenses associated with hiring additional data collectors to support our study – made it more practical to work with a smaller, concentrated sample. The logistics of interacting with a vast number of participants, especially via phone-based surveys conducted during working hours, further justified our approach. This method not only streamlined our data quality checks but also expedited the results, a crucial factor given the ongoing pandemic. Our choice of the 366 participants was methodically made using randomized sampling, ensuring they accurately represented the broader affected group's characteristics. In essence, our goal was to harmonize the acquisition of dependable and prompt data with the efficient use of our resources.

**2. As you mentioned and is agreed on, the post COVID syndrome Sx should be lasting for at least 2 months post acute infection but WITH NO ALTERNATIVE DIAGNOSIS FOUND, how did you make sure of that?**

Since our staff are insured, the COVID-19 team was actively monitoring them throughout their infection and subsequent recovery. We consistently urged them to report any lingering symptoms and to consult specialists if these symptoms continued to impact their quality of life. Concurrently, the medical history gathered during our study was comprehensive enough to confirm that the enduring symptoms they reported were indeed new and consistent with post-COVID-19 effects.

**3. Regarding the post COVID Sx that were found associated with obesity, many of them are actually related to OSA symptoms, do you have any clarification?**

While obesity is a known risk factor for both post-COVID symptoms and obstructive sleep apnea , it's vital to distinguish between the two given their symptom overlap. In our study, we differentiated symptoms unique to post-COVID syndrome, such as anosmia or ageusia, from those commonly seen in OSA. By examining participants' prior medical histories and, we could discern the origin of certain symptoms. We also assessed the timing of symptom onset, considering whether issues like fatigue predated a COVID-19 infection.

**4. Your discussion of the results is superficial and doesn't give possible explanations for the association you found; you only mention evidence from literature about your results.
You don't need to repeat your results in details especially numbers in the discussion section.**

I update this section by adding the followings:

- The symptomatic profile we observed, dominated by fatigue, smell impairment, and fever, resonates with international findings but with certain nuances. Such variations could stem from differences in genetic predispositions, viral strains, or environmental factors, warranting further investigation.

- While it's recognized that post-COVID symptoms might be influenced by factors like age, gender, or comorbidities, our study interestingly identified associations with smoking. This aligns with some international studies but diverges from others, hinting at potential genetic, behavioral, or regional factors at play.

Many thanks for your valuable comments.

We are looking forward to hearing from you.
